# Effectiveness of lenalidomide in relapsed primary cutaneous diffuse large B‐cell lymphoma, leg type

**DOI:** 10.1002/ccr3.2137

**Published:** 2019-04-05

**Authors:** Mahdi Al Dhafiri, Flore Sicre de Fontbrune, Eduardo Marinho, Lydia Deschamps, Julie Di‐Lucca, Beatrice Crickx, Vincent Descamps

**Affiliations:** ^1^ Department of Dermatology Bichat Hospital Paris France; ^2^ Department of Dermatology, College of Medicine King Faisal University Al‐Ahsa Saudi Arabia; ^3^ Department of Hematology Saint‐Louis Hospital Paris France; ^4^ Department of Anatomopathology Bichat Hospital Paris France; ^5^ Department of Dermatology Lausanne University Hospital Lausanne Switzerland

**Keywords:** chemotherapy, leg type, lenalidomide, primary cutaneous diffuse large B‐cell lymphoma

## Abstract

The primary cutaneous diffuse large B‐cell lymphoma, leg type (PCDLBCL‐LT) has a poor prognosis. R‐CHOP with or without radiotherapy is the available recommendations for first‐line treatment. Relapses/refractory cases are frequent with no standardized therapeutic guidelines. Lenalidomide seems to be an excellent therapeutic option as a second‐line treatment of relapsed PCDLBCL‐LT.

## 1 INTRODUCTION

Primary cutaneous diffuse large B‐cell lymphoma, leg type (PCDLBCL‐LT) is one of the major four subtypes of cutaneous B‐cell lymphoma 1 and characterized by its aggressive nature, poor prognosis, and occurrence in advanced age, with five‐years survival estimations of approximately 50%‐60%. 2, 3

Clinically it is represented as red to purple nodules that arise mainly in the lower extremities. Histologically it is characterized by the presence of confluent sheets of large cells with round nuclei, with strong expression of Bcl‐2, as well as intermediate or positive staining for MUM‐1 protein.^1^, ^4^, ^5^

The initial treatment is based on rituximab associated with polychemotherapy,^2^, ^3^, ^6^ with no ideal therapy for relapsing or refractory forms. To date, there are two previously reported cases showing a promising effect of lenalidomide in relapsed cases,^6^, ^7^ with one recent phase II study on the efficacy of lenalidomide in relapsing or refractory PCDLBCL‐LT.^4^

Herein, we exhibit a case successfully treated with lenalidomide (Revlimid®) in second‐line treatment.

## 2 CASE REPORT

**Figure 1:**
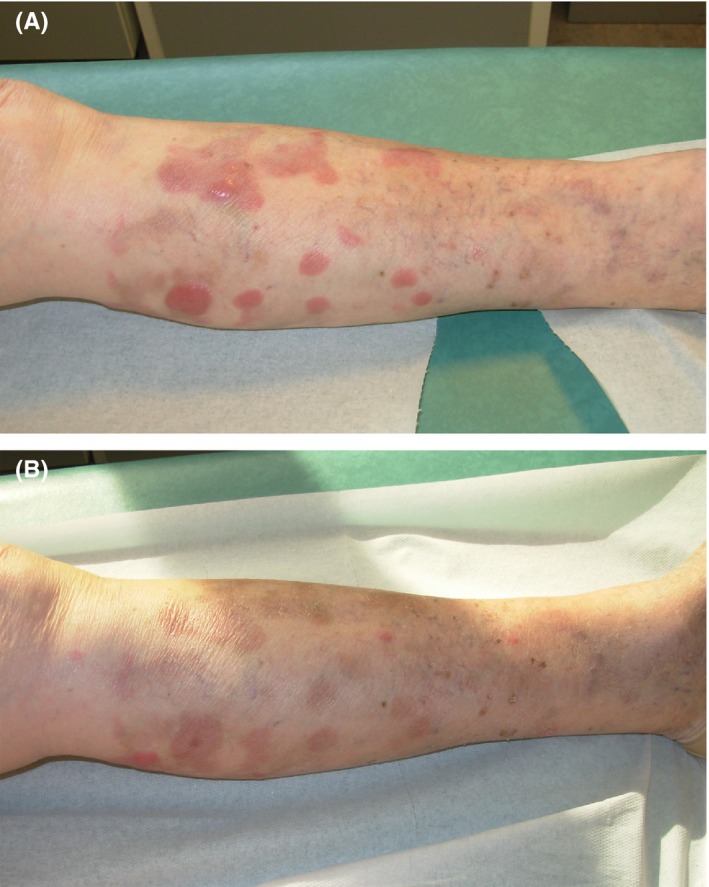
A, Progressing lymphoma at left leg with multiple growing tumors. B, Cutaneous relapse after second R‐CHOP cycle

**Figure 2:**
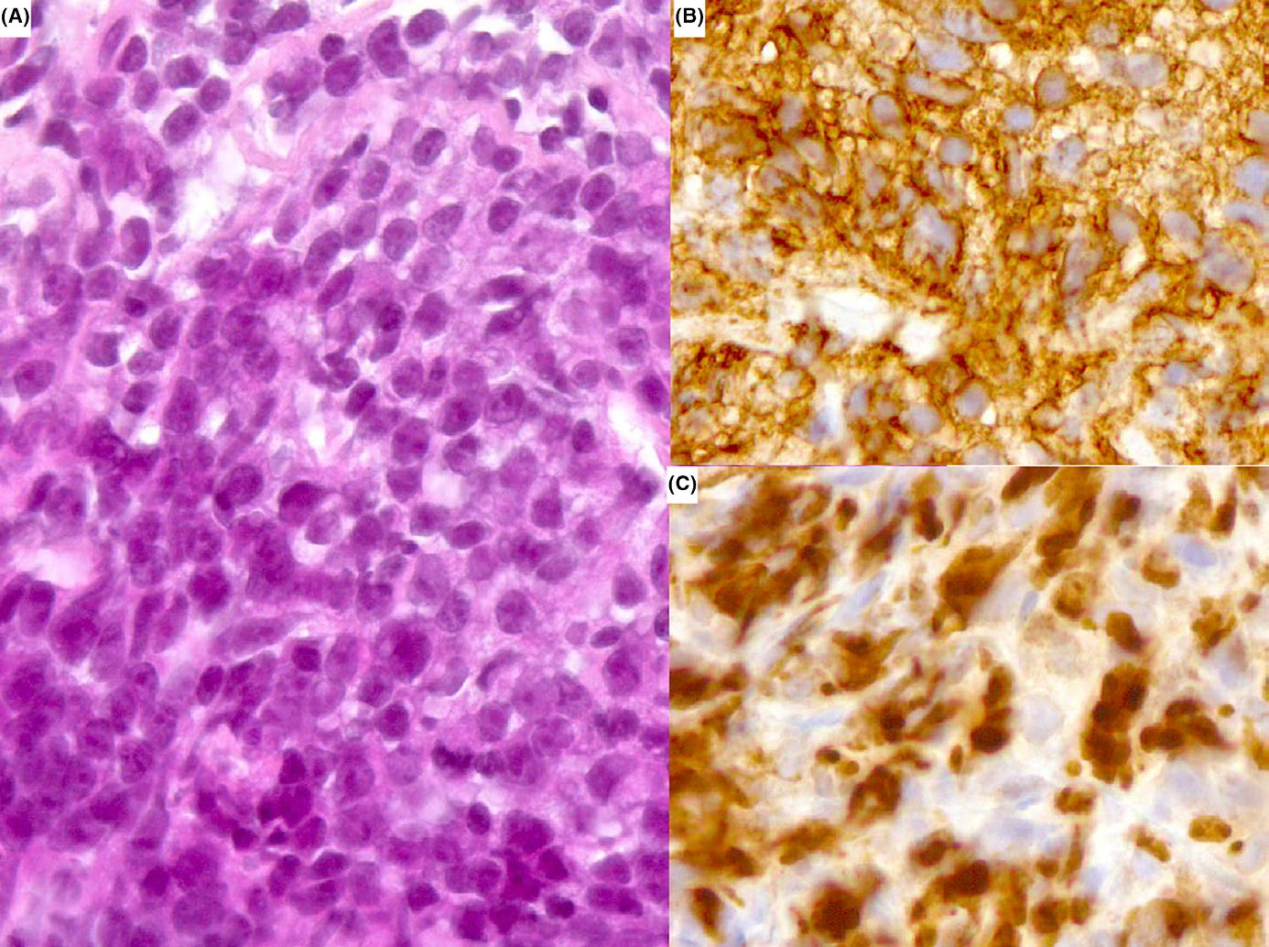
Histological examination of a biopsy of the mediastinal mass (original magnification × 400): numerous large cells with predominant immunoblasts (A) with CD20+ (B), and Ki‐67 (C) staining

An 80‐year‐old female patient was referred to our hospital for a relapse of PCDLBCL‐LT. She was previously treated by systemic immunochemotherapy with rituximab, cyclophosphamide, doxorubicin, vincristine, and prednisone (R‐CHOP). The first cutaneous lesions of her left leg developed on March 2009. Initial screening by total body computed tomography (CT) showed, other than the multiple nodules of the left leg (Figure 1A), a mediastinal mass of 70 × 37 mm associated with pleural effusion. The initial histological investigations confirmed the diagnosis of PCDLBCL‐LT in both skin lesions and mediastinal mass, with the positivity of CD20, Ki67, and bcl2 (Figure 2). After the completion of 5 cycles of treatment, the clinical examination, and total body CT scan showed complete control of PCDLBCL‐LT on June 2010 with clearance of the cutaneous and mediastinal lesions. However, the patient developed neutropenia, and the sixth cycle was not performed.

The patient then developed a relapse of both cutaneous and mediastinal lesions in September 2010 (Figure 1B). The second course of R‐ CHOP did not control the disease and was responsible for hematological toxicity, including severe neutropenia. Lenalidomide (10 mg/day for 21 days of every 28‐day cycle) was proposed on November 2010, associated with a dexamethasone 20 mg on days 1‐7‐14 monthly.

This treatment was well tolerated and permitted a good control with a complete disappearance of the cutaneous nodules with control of 5 months of her lymphoma. The lymphoma then progressed by the 6th month (on May 2011) with a rapid progression of the mediastinal mass. She deceased on June 2011.

## 3 DISCUSSION

PCDLBCL‐LT is rare non‐Hodgkin's lymphoma (2.6% of primary cutaneous non‐Hodgkin's lymphomas), affecting elderly people (median age of 76 years),^1^ and known by its aggressive behavior and poor prognosis. It is represented by solitary or multiple rapidly growing nodule localized mainly on lower limb unilaterally or bilaterally. Ulceration of these lesions is possible.^2^, ^3^, ^4^ Unlike other primary cutaneous B‐cell lymphomas, secondary extracutaneous dissemination is common,^3^, ^4^ including lymph nodes, central nervous system, bone, and liver.^1^, ^8^ PCDLBCL‐LT is characterized by the positivity of MUM‐1, CD20, and high Bcl‐2 expression with lack of CD10.^2^, ^9^ Dual expression of both bcl‐2 and c‐myc is also common and associated with inferior overall survival.^3^, ^10^ However, up to 10% of PCDLBCL‐LT presents lack of bcl‐2 and Mum‐1 staining.^1^ Genetically, NF‐κB pathway‐activating mutations were observed in PCDLBCL‐LT, and this includes *CD79B*, *CARD11*, and the mutation of *MYD88* which was confirmed to be the most prevalent mutation (~75%).^3^, ^9^, ^11^ Moreover, *MYD88* mutation can be used as a diagnostic feature to differentiate PCDLBCL‐LT from the primary cutaneous follicular large B‐cell lymphoma.^9^

The main adverse prognostic factors include multiple lesions, old age, *MYD88* mutation, advanced T stage of the tumor, and leg localization of the lesion.^2^, ^4^, ^5^ First‐line treatment in the diffuse or multifocal form of PCDLBCL‐LT is as in diffuse large B‐cell lymphomas (DLBCL)^3^ depends on polychemotherapy associating rituximab (R‐CHOP) with or without radiotherapy.^12^, ^13^ This combined therapy could be replaced by less‐aggressive treatment of rituximab and polychemotherapy (R‐PCT) in oldest or frailest patients.^2^, ^4^ However, for the solitary lesion, radiotherapy should be considered as a first‐therapeutic choice. It could also be treated by surgical resection.^8^, ^12^, ^13^ Additionally, it has been observed that the 5‐year survival rate has been increased by about 65% to 75% over time since the use of rituximab‐PCT with or without anthracyclines.^2^ Nevertheless, neutropenia and consecutive infection are the main adverse effect and cause of morbidity and mortality in this treatment.^2^

Spontaneous remission of PCDLBCL‐LT is extremely rare, and it was reported in five cases.^1^, ^14^, ^15^ The cause of this spontaneous regression in unknown, and it has been described as a result of probable response of immune system to bacterial or viral infection, or traumatic causes including biopsies, or apoptosis or particular condition of the tumor microenvironment as well.^1^, ^14^, ^17^ All biopsies taken after regression of the published cases showed superficial and deep dermal inflammatory T‐cell infiltrate suggesting that un inadequate T‐cell immune response may play a role in the disease pathogenesis.

Refractory cases to R‐PCT are possible, and rapid recurrence after initial treatment is still frequent and remains a challenging issue due to lack of standardized therapeutic protocol.^2^, ^4^, ^7^ Lenalidomide (Revlimid®) is a derivative of thalidomide,^18^ and it is an oral immunomodulatory agent with multiple mechanisms of action that interfere with the growth of aggressive non‐Hodgkin's lymphomas through alteration of the tumor microenvironment and enhancing the cytotoxic activity of T cells and natural killer cells.^19^ It performs an inhibition of cell signaling engaging NF‐κB and IFN‐β, through its antiproliferative and antiangiogenic effects.^4^

Lenalidomide was approved in December 2005 by the FDA for the treatment of red blood cell transfusion‐dependent anemia due to myelodysplastic syndrome (MDS) associated with a chromosome 5q31 deletion. It is also indicated in the treatment of other conditions, including plasma cell malignancy, mantel cell lymphoma, cutaneous T‐cell lymphoma, and multiple myeloma.^20^ The most common adverse events are neutropenia and thrombocytopenia.^19^

The efficacy of lenalidomide was demonstrated on relapsing and refractory DLBCL, and it looks to be a good candidate for PCDLBCL‐LT.^4^ Thus its effectiveness was discussed previously in two case reports of relapsed PCDLBCL‐LT, showing partial remission of the disease with excellent tolerance of the treatment in an 83‐year‐old woman.^6^ And the complete resolution was obtained after combined therapy of lenalidomide with rituximab in a 78‐year‐old woman.^7^ The efficacy of single‐agent lenalidomide in relapsed/refractory PCDLBCL‐LT was recently discussed in a small phase II study (n = 19), and the 6‐month overall response rate was 26%. However, the response was significantly higher with the absence of the *MYD88* mutation.^4^

This observation underlines the interest of the association lenalidomide‐dexamethasone in the management of PCDLBCLs‐LT.

## 4 CONCLUSION

PCDLBCL‐LT has a poor prognosis with no standardized treatment recommendations available in relapsed forms. The adverse therapeutic effects are most likely related to the advanced age and poor general condition of patients. A combined lenalidomide therapy, as well as more targeting monotherapy, deserves to be evaluated as a second‐line treatment for this affection.

## CONFLICT OF INTEREST

None declared.

## AUTHOR CONTRIBUTION

MA: provided scientific and bibliographic feature and wrote the paper. FSDF: participation in the therapeutic plan and patient monitoring. EM&LD: provided histopathologic work‐up. JDL&BC: provided clinical follow‐up and control of the patient as well as participation in the therapeutic plan and evaluation. VD: supervision and involvement in the scientific work‐up and writing the paper, as well as patient follow‐up.
